# Biometric authentication data with three traits using compression technique, HOG, GMM and fusion technique

**DOI:** 10.1016/j.dib.2018.03.115

**Published:** 2018-03-31

**Authors:** Balaka Ramesh Naidu, Maddali Surendra Prasad Babu

**Affiliations:** a Department of Information Technology, AITAM Engineering College, Tekkali, India; b Department of Computer Science & Systems Engineering, Andhra University, Vizag, India

## Abstract

This paper presents a three trait identification model called multimodal recognition system developed by using different traits like face, finger and voice (Babu and Naidu, 2014, 2016; Balaka and Surendra, 2017) [1–3]. This system provides more security when compare to existing works. Initially, all the traits are followed by pre-processed, extract features using Histogram of oriented gradients (HOG), then apply Gaussian mixture model (GMM) for finding probability density function (PDF) values and then combining these features by using Score level fusion. The result of these features considered as a trainee dataset. In verification process, each test image trait compare with the trainee dataset. This entire process of authentication is done by using machine learning based technique.

**Specifications Table**

**Table:** 

Subject area	Image Processing
More specific subject area	Compression, Feature Extraction, Bio-metric Authentication with multiple traits
Type of data	Facial & Fingerprint Images, Audio files, Tables, Figures.
How data was acquired	Traits are acquired with sensor and processed with HOG and GMM.
Data format	Tiff data, jpeg, audio data, authentication data
Experimental factors	Three traits: Face, Fingerprint and Audio format are targeted. Compression techniques, HOG, GMM and fusion on three traits. 150 Face, Finger and Audio format to be used in authentication purposes are extracted with this approach.
Experimental features	Extract three different biometric traits from each person. All the 150 traits showed that there are biometric recognition similarities.
Data source location	AITAM,TEKKALI, ANDHRA PRADESH, INDIA
Data accessibility	The data is provided with this article

**Value of the data**

● The data presented in this article are face, fingerprint and audio format are obtained from AITAM, TEKKALI. These are subjected to Compression, HOG, GMM and fusion has shown the high specificity.
● These data suggest that THREE traits are useful and robust method for providing information for various detections.
● Access to the raw face, fingerprint and audio format data allows researchers to perform further analysis based on their own computational algorithms.

## 1 Data

**Fig. 1:**
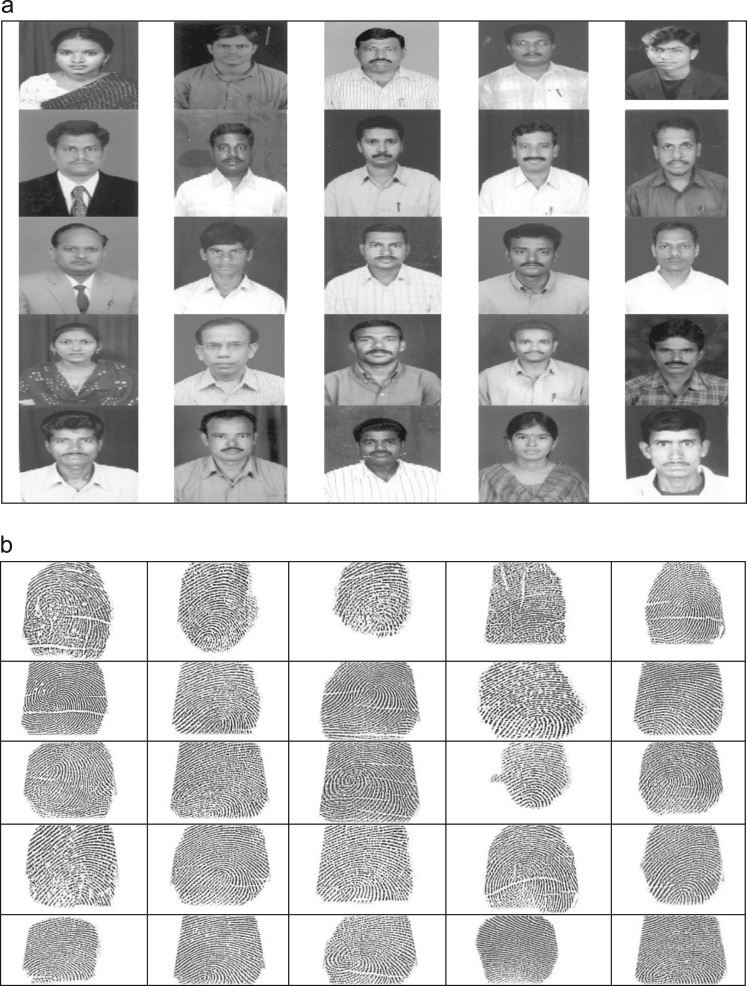

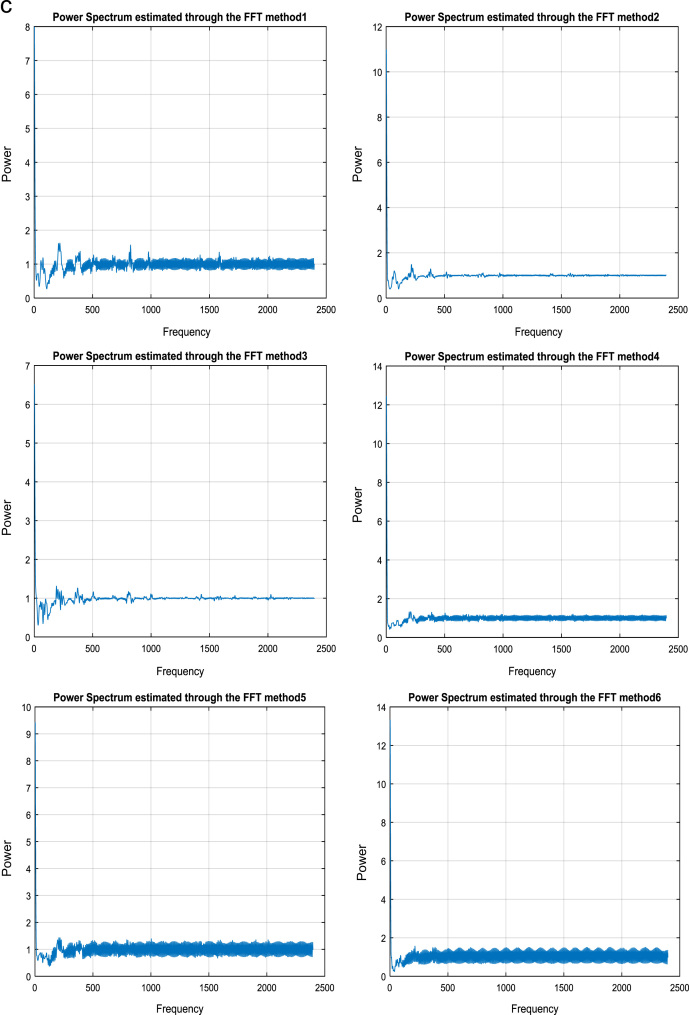

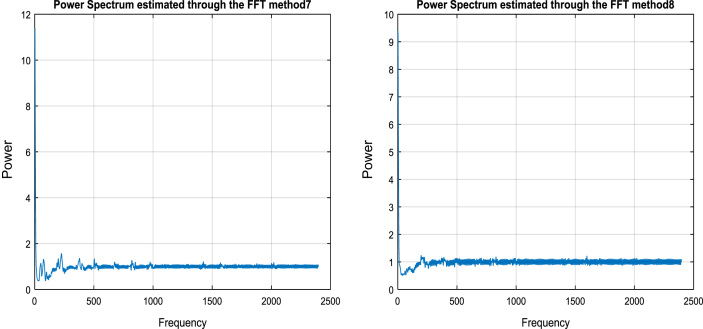
(a) Face dataset. (b) Fingerprint dataset. (c) Voice dataset.

In this paper, consider the AITAM college dataset of three traits face, fingerprint and voice for experiments. The entire recognition system can be divided into five steps. In the step 1 collect various face images from AITAM College information shown in Fig. 1(a), respective fingerprints and voice signals are shown in Fig. 1(b) and (c). Complete dataset of AITAM College is presented in Appendix Table 1. In this article 150 face, finger and voice traits are collected. Initially, The finger data is collected using SecugenHamster Plus fingerprint scanner which gives images of size 260×300 pixels. These images are again compressed to 69×57 pixels without losing predominant information to match with the sizes of facedata. The face data is collected with single image size of 580×560 pixels and then compressed to 69×57 pixels without losing predominant information as database management is also very important. The voice is collected from the same personswith audio length of 5 secs and sampling frequency of 48 KHz. Then it is downsampled to 5 times to reduce the database size. This voice is denoised and itspower spectrum is obtained along with dominant frequency points. The data is taken from the staff members of AITAM Engineering College located in Tekkali, India. In this paper face and finger data is presented in Appendix I and voice data link is presented in Appendix II. The authentication system using face recognition is there since long time but the authentication system can be break down in some cases using morphing etc. So to make the system more secure, along with face recognition, fingerprints and voice are considered. Dealing with these three traits separately is common practice. In this paper, all these traits are converted into single entity using fusion techniques and treated it as final data for authentication. With this, the database can be utilized in more efficient way and the authentication system will become more robust. For this, the fingerprints and voices are collected from the same persons [1], [2], [3]. Only 3933 samples of the total audio signal is considered to match with the sizes of face and finger. The images considered here are in Tiff format and audio is in wav format. As it is difficult to present all the data, only few samples are presented in this article.

The face and fingerprint traits shown in Fig. 1(a) and (b) are pre-processed by different steps such as convert RGB to gray, remove noise and compressed using standard techniques PCA, KLT, DCT, HAAR. Fig. 1(c) is different voice signals of eight persons.

In the second step, extract the features of each trait. This is achieved by Histogram of oriented gradients. In this, cells can be either radial or rectangular shape and these are spread over 0–360° or 0–180° and it depends on whether the gradient is signed or unsigned.

**Fig. 2:**
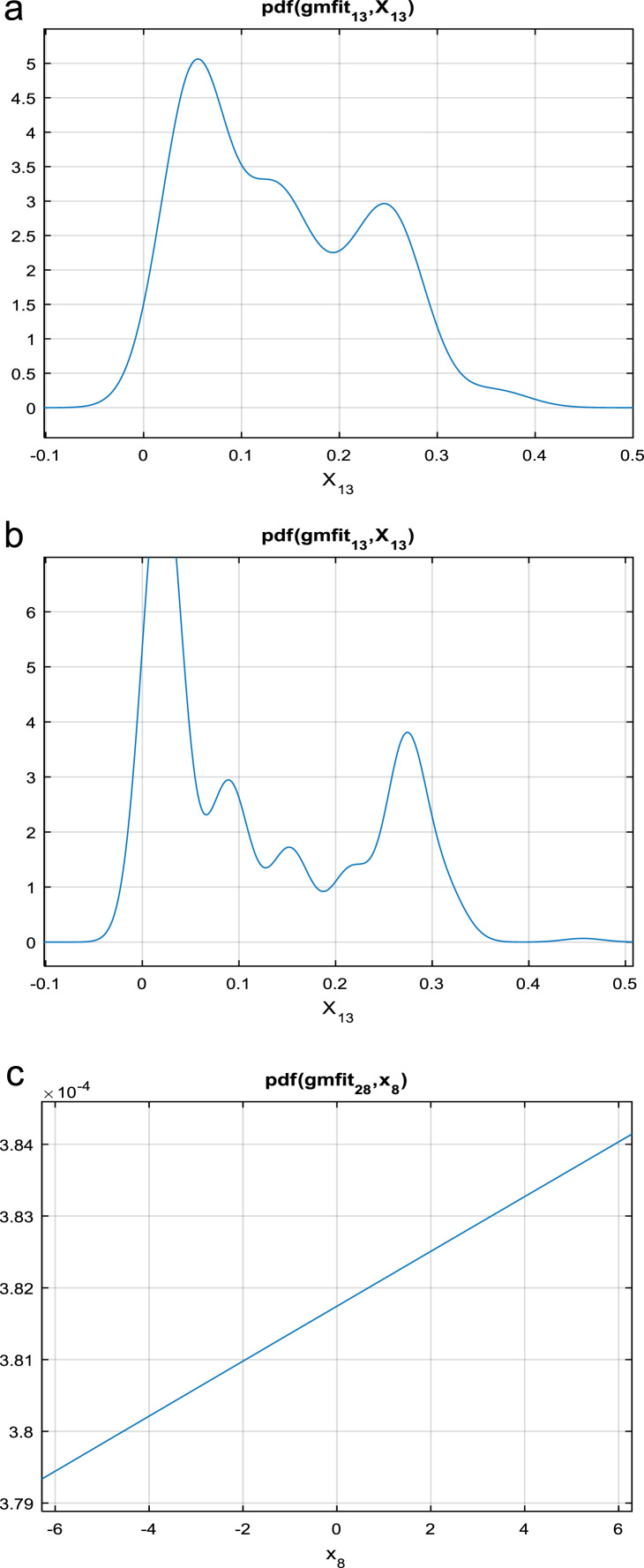
(a) GMM face. (b) GMM fingerprint. (c) GMM for voice.

In the third step, the Gaussian mixture model is applied for finding the Gaussian distribution for face shown in Fig. 2(a), fingerprint shown in Fig. 2(b) and voice shown in Fig. 2(c).

**Fig. 3:**
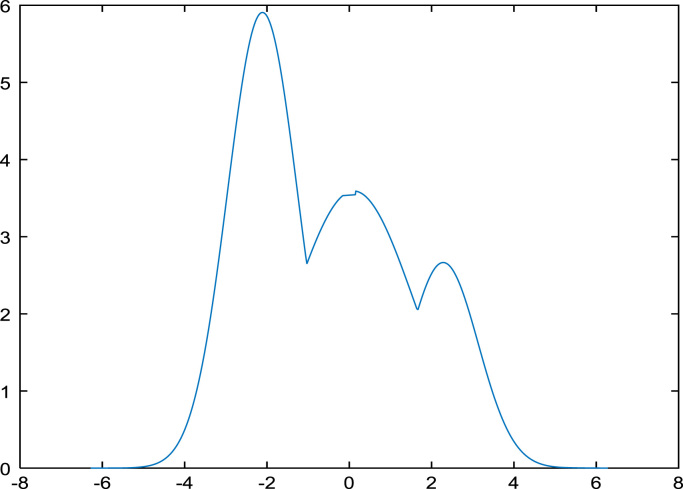
An example of after fusion of face, fingerprint and voice.

In the fourth step, combine the fingerprint, face and voice traits of PDF values by using score level fusion technique. This dataset is called training dataset (Fig. 3).

In the fifth step, test dataset is compared with the training dataset by using correlation method. If match is found then it is “accepted” else “not accepted”. This is achieved by following Eq. (1).

(1) $$\rho_{X,Y}=\frac{\left[\left(X-\left(\mu X\right)\right)\left(Y-\left(\mu Y\right)\right)\right]}{\sigma X\sigma Y}$$

Where μ is mean, Let us assume, A, B are two different image vectors. In the step1, calculate the mean of A ((i.e.,μX) and B (i.e.,μY). In the step2, subtracts the mean value from the A matrix (i.e., A−μX) and subtracts the mean value from the B (i.e., B−μY). In the step 3, multiply both A_sub and B_sub and then calculate the mean value (i.e., Cov_AB=mean (A_sub×B_sub)). In the step 4, find standard deviation of both A and B. Finally, $\rho_{X,Y}=\frac{\mathrm{CovAB}}{\mathrm{StdA}X\mathrm{StdB}}$. If correlation coefficient result is “1” then highly correlated else “0” if it is not correlated.

## 2 Experimental design, materials and methods

The present model is designed to retrieve the quality of output image trait based on the principle is as follows: pre-processing which is discussed in Section 2.1 and extract features using HOG which is discussed in Section 2.2, Gaussian mixture model (GMM) process which is discussed in Section 2.3 is performed to predict the probability density values (PDF), Fast Fourier Transform for converting audio signal to digital which is discussed in Section 2.4, score level fusion (SCL) combines all the features of each individual traits which is discussed in Section 2.5. All these steps are implemented to generate a training dataset. To test the results, we consider the testing dataset such as face, fingerprint and voice compared with existing training dataset. Testing dataset is also generated by the same process as discussed. Using correlation both the trainee and test datasets are compared. Finally the result shows “user is authenticated as a genuine user” or “user is not genuine”.

### 2.1 Image pre-processing

**Fig. 4:**
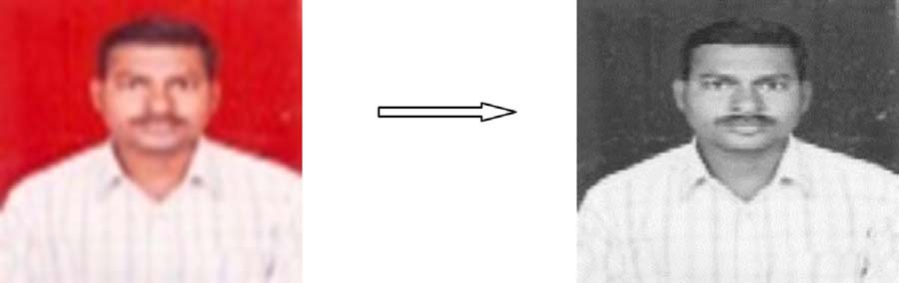
Convert RGB to gray.

**Fig. 5:**
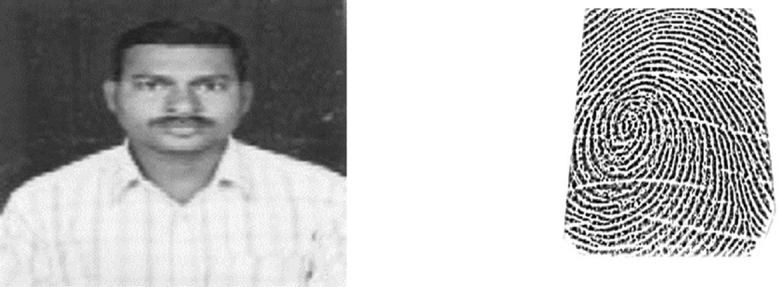
Result of filter.

**Fig. 6:**
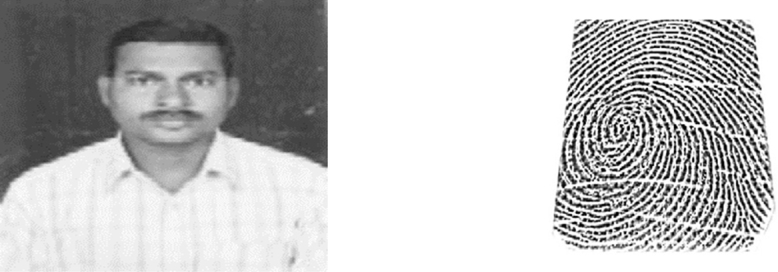
Result of compression.

The image pre-processing assessment is used to convert the RGB to Gray level shown in Fig. 4, removal noise shown in Fig. 5 and compression processes applied to digital images and result is shown in Fig. 6. In the context of image pre-processing, for example, such kind of assessment is used to improve the quality of the reconstructed image. In this paper pre-processing image quality assessment metrics are divided into three categories:

1) RGB to Gray level quality, where the target image is converted to tiff image format by using rgb2gray() method in MATLAB.
2) Apply median filter for removing noise in images; and
3) Apply lossy compression technique on both face and finger for removing reluctant information in image. In this work, we propose the different standard compression methods like DCT, HAAR and KLT.

After removing the noise from the traits, then apply the compression technique which is widely used to reduce the storage space in the image. In this paper we propose the different compression methods like DCT, HAAR, KLT and PCA.

### 2.2 Histogram of oriented gradients (HOG)

**Fig. 7:**
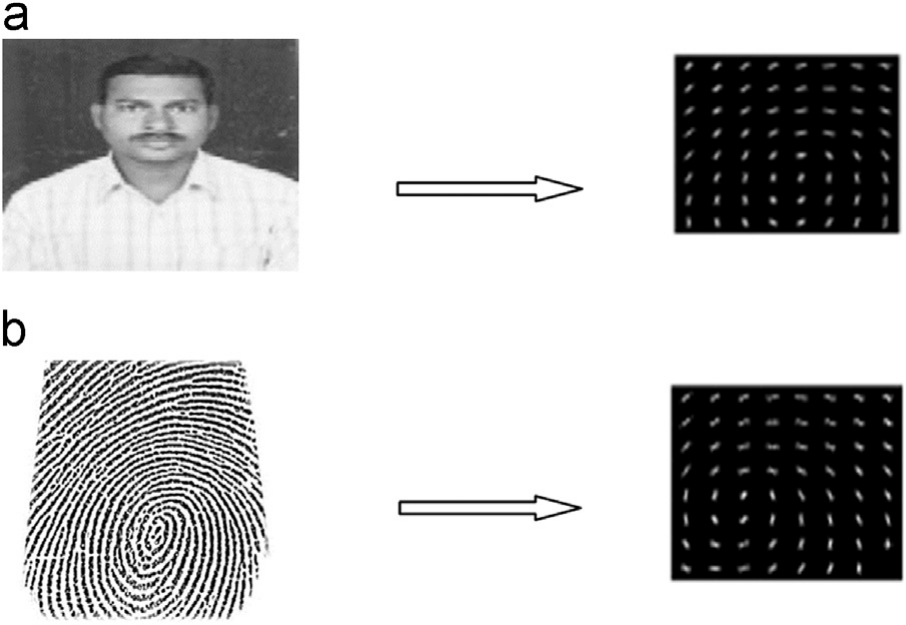
(a) HOG features of face, (b) HOG features of fingerprint.

HOG implementation is based on four steps [4], [5]. These include Gradient calculation, Histogram of Gradients, Block normalization and Feature vector. The following steps for calculating the HOG descriptor for a 64×128 image are listed in Fig. 7(a) and (b).

**Step1**: Gradient calculation: To calculate a HOG descriptor, first need to calculate the x horizontal and y vertical gradients, after calculation of all gradients, then calculate the histogram of gradients. This is easily achieved by filtering the image with the following kernels masks are 1×3 and 3×1

**Step 2**: Calculate histogram of gradients: In this step, the image is divided into 8×8 cells and a histogram of gradients (64 magnitudes and 64 directions i.e. 128 numbers) is calculated for every 8×8 cells. Histogram of these gradients will provide a more useful and compact representation and also very less noise. Convert these 128 numbers into a 9-bin histogram which can be stored as an array of 9 numbers.

**Step 3**: Block normalization: In the previous step, we created a histogram based on the gradient of the image. Gradients of an image are sensitive to overall lighting. If you make the image darker by dividing all pixel values by 2, the gradient magnitude will change by half, and therefore the histogram values will change by half. Ideally, we want our descriptor to be independent of lighting variations. In other words, we would like to “normalize” the histogram so they are not affected by lighting variations.

**Step 4**: Calculate the HOG feature vector: To calculate the final feature vector for the entire image patch, the 36×1 vectors are concatenated into one huge or massive vector. Size of the vector is calculated using step 4.1 and 4.2.

**Step 4.1**: In this step finds the number of block positions, for example the 16×16 blocks there are 7 horizontal and 15 vertical positions making a total of 7×15=105 positions.

**Step 4.2**: Each one (16×16 block) is represented by a 36×1 vector. Finally, combine all the blocks into one huge vector is a 36×105=3780 dimensional vector.

### 2.3 Gaussian mixture model (GMM)

Gaussian is typical symmetric bell shaped curve that is exactly similar parts facing each other or around an axis. Mixture model is probabilistic which assumes the underlying data to fit in to a mixture distribution. GMM is parametric representation of PDF based on sum of weighted multi variant Gaussian distributions. This model commonly used in continuous measurements or features in biometric traits.GMM used in classification, signal processing, speaker recognition, Language identification and so on. In this paper, we propose Gaussian Mixture model is considered since most of the traits like facial, fingerprint and voice templates exhibit a pattern, which in nature resembles a normal distribution [6], [7]. The following mathematical notation of Gaussian mixture model is (shown in Eq. (2)).

(2) $$P\left(x\right)=W_{1}P_{1}\left(x\right)\;+\;W_{2}P_{2}\left(x\right)\;+\;W_{3}P_{3}\left(x\right)\;+\;\ldots \ldots \ldots \;+\;W_{n}P_{n}\left(x\right)$$

Where *P*(*x*) is mixture component, *W*_1*,*_ *W*_2_*, W*_3_…… *W*_*n*_ is mixer weight or coefficient and *P*_*i*_(*x*) is density function where *i*=1, 2, 3…*n*.

The most common distribution is the Gaussian (Normal) density function in which each of the components are the Gaussian distributions, each one with their own mean and variance parameters (shown in Eq. (3)).

(3) $$P\left(x\right)=W_{1}N(x|\mu_{1}\underset{1}{\sum })\;+\;W_{1}N(x|\mu_{2}\underset{2}{\sum })\;+\;W_{1}N(x|\mu_{3}\underset{3}{\sum })\;+\;\ldots \ldots \;+\;W_{1}N(x|\mu_{n}\underset{n}{\sum })$$

where $\mu_{1}$, $\mu_{2},\mu_{3},\ldots \mu_{n}$ are means, $\Sigma_{1}\;,\Sigma_{2},\Sigma_{3}\ldots .\Sigma_{n}\mathrm{are}$ covariance matrix of individual components (PDF) and P_i_(x) is result of density functions. The function of the Gaussian distribution is (shown in Eq. (4)):

(4) $$f\left((,x,)\right)=\frac{1}{\sqrt{2\pi \sigma^{2}}}e^{-\frac{(x-\mu )2}{2\sigma^{2}}}$$

*μ* is the mean or expectation of the distribution, *σ* is the standard deviation and *σ*^2^ is *π* = 3.14151 variance and *e*=2.1728.

The formula of *Z* score is (shown in Eq. (5)):

(5) $$Z=\frac{x-\mu }{\sigma }$$

*Z* is the "*z*-score" that is (Standard Score) and x is the value to be standardized. A Gaussian mixture model is a weighted sum of *M* component Gaussian densities as given by the Eq. (6),

(6) $$p\left(x|\lambda \right)=\sum_{i=1}^{M}w_{i}g\left(\;\frac{x}{\mu_{i}},\underset{i}{\sum }\right)$$

where x is a D-dimensional continuous valued data vector (i.e. measurements or features), $w_{i}$ *, i*=1*…M*, are the mixture weights, and *ɡ* (*x|μ*_*i*_*,* Σ_i_), *i*=1*… M*, are the component Gaussian densities. Each component density is a D-variant Gaussian function of the form is (shown in Eq. (7)),

(7) $$g(\left(x|\mu_{i},\underset{i}{\Sigma }\right)=\frac{1}{{\left(2\pi \right)}^{\frac{D}{2}}|\underset{i}{\Sigma }{|}^{\frac{1}{2}}}\exp\left\{-\frac{1}{2}\left(x-\mu_{i}\right)\prime \sum_{i}^{-1}\left(x-\mu_{i}\right)\right\}$$

With mean vector µ_i_ and covariance matrix Σ_i_, the mixture weights satisfy the constraint that the $\;\sum_{i=1}^{M},w_{i}=1$. The complete Gaussian mixture model is parameterized by the mean vectors ($\mu_{i})$, covariance matrix (Σ_i_) and mixture weights ($w_{i})$ from all component densities.

### 2.4 Fourier transforms representation for voice signal

In this paper, develop a speaker recognition technology is used to find the dissimilarity among speakers and speech signals [8]. Generally, voice have individual trait for every person, therefore to recognize the person by their voice is more helpful technique in recent trends. This is achieved by Fast Fourier transform. Spectrum analysis of a ‘speech signal’ is the method of shaping the ‘frequency domain representation’ of a time domain signal and this method usually known as Fourier transform. The Discrete Fourier Transform (DFT) is used to find out the frequency content of analog signals. And Fast Fourier Transform (FFT) is a competent process for calculating the DFT.

The Fourier transform of the function f(t) is the function F(ω) where

(8) $$F\left(\omega \right)=\int_{-\infty }^{\infty }\;f\left(t\right).e^{-j\omega t}\mathrm{dt}$$

and the inverse Fourier transform is

(9) $$f\left(t\right)=\frac{1}{2\pi }\int_{-\infty }^{\infty }F\left(\omega \right).e^{\;j\omega t}d\omega$$

If f(t) is considered as a signal then F(ω) is the signal's spectrum when a signal is discrete and periodic, we use discrete Fourier transform. Suppose our signal is a_n_ for *n*=0, 1, …, *n*−1. The discrete Fourier transform of a, also known as the spectrum of 'a' is

(10) $$A_{k}=\sum_{n=0}^{N-1}e^{-j\frac{2\pi }{N}kn}a_{n}$$

This can be written

(11) $$A_{k}=\sum_{n=0}^{N-1}W_{N}^{kn}a_{n}$$

$$W_{N}=e^{\;j\frac{2\pi }{N}}$$

and $W_{n}^{k}$ for *k*=0, …, *N*−1 are called *N*th roots of unity. The sequence *a_n_* is the inverse discrete Fourier transform of the sequence $A_{k}$.

The formula for inverse DFT is

(12) $$a_{n}=\frac{1}{N}\sum_{n=0}^{N-1}W_{N}^{-kn}A_{k}$$

The FFT is a fast algorithm for computing the DFT. To compute the DFT of an N-point sequence would take O (*N*^2^) multiplies and adds. The FFT algorithm computes the DFT using O (NlogN) multiplies and adds.

### 2.5 Fusion

Fusion means combining different images into a single image with more informative than any of the individual input images and also suitable for both visual perception and further computer processing. In this paper, we combine three feature values such as face, finger print and voice. The solution to multimodal biometrics is the fusion of the different biometric data after feature extraction. Fusion can be implemented in three types like score level fusion, feature level fusion and decision level fusion [9]. In this paper we propose score level fusion is considered to fuse the biometric traits. Fusion of three traits results are presented in Appendix III from Fig. 8(a)–(h).

#### 2.5.1 Score level fusion

Score level Fusion is used for finding the matching scores between different biometric traits [10]. The scores in biometric system may be similar or dissimilar. In score level fusion, the image is reduced into single piece as a match score or similarity score by a classifier and that classifier trains and test the input data. Trained and tested data are compared to find the required biometric trait. Let us consider (X_ij_,Y_ij_), (X_ik_,Y_ik_) and (X_il_,Y_il_) as pixels of three different images, where i indicate position of pixel and *j*, *k*, *l* indicates image number. Initially we compare (X_ij_,Y_ij_) and (X_ik_,Y_ik_ ) image vectors and a new fused image (X_im_,Y_im_) is formed based on the following condition. If X_ij_ > X_ik_ then X_im_= X_ij_ else X_im_= X_ik_.

If Y_ij_ > Y_ik_ then Y_im_ = Y_ij_ else Y_im_ = Y_ik_ In this case the maximum value among the pixels is considered as score. Secondly we compare (X_im_,Y_im_) and (X_il_,Y_il_) image vectors, a new fused image (X_n_,Y_n_) is formed based on the following condition. If X_im_ > X_il_ then X_n_ = X_im_ else X_n_ = X_il_ If Y_im_ > Y_il_ then Y_n_ = Y_im_ else Y_n_ = Y_il_.
