# Supplementary material for: Biometric authentication data with three traits using compression technique, HOG, GMM and fusion technique
Source: Data Brief. 2018 Mar 31;18:1976–86. doi: 10.1016/j.dib.2018.03.115 (PMC5996745; doi:10.1016/j.dib.2018.03.115)
Supplement: Supplementary file 1 — Supplementary material. [file mmc1.docx]

authors not having any conflict of interest
