# Supplementary material for: Biometric authentication data with three traits using compression technique, HOG, GMM and fusion technique
Source: Data Brief. 2018 Mar 31;18:1976–86. doi: 10.1016/j.dib.2018.03.115 (PMC5996745; doi:10.1016/j.dib.2018.03.115)
Supplement: Supplementary file 2 — Supplementary material. [file mmc2.pdf]

APPENDIX I

TABLE 1 AITAM COLLEGE DATA SET - FACE & FINGER

Face	Thumb		Face	Thumb
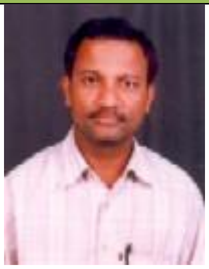	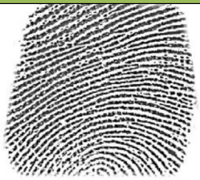		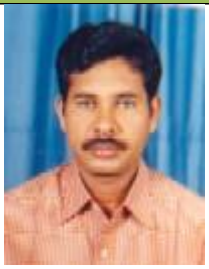	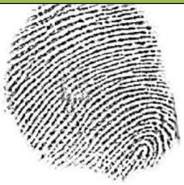
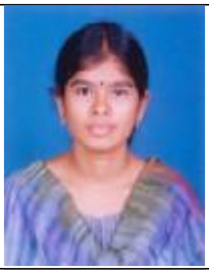	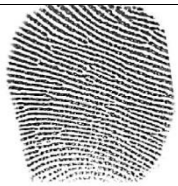		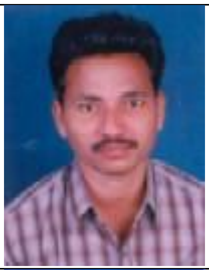	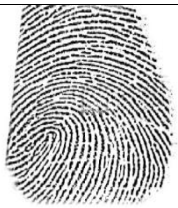
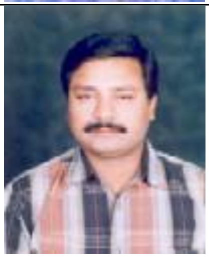	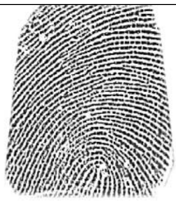		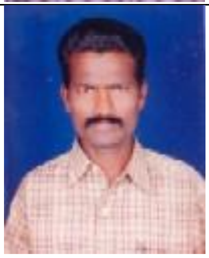	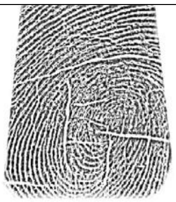
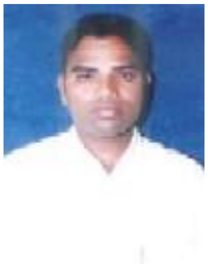	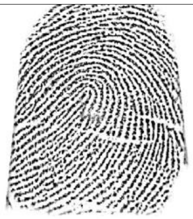		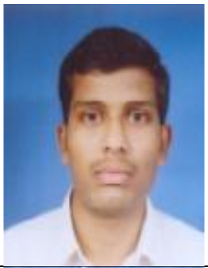	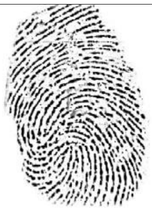
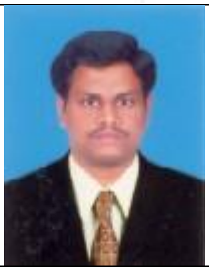	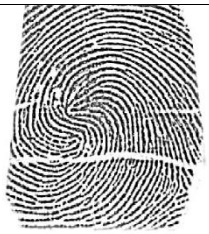		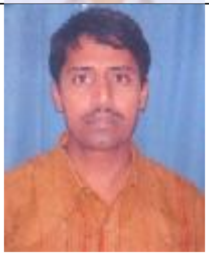	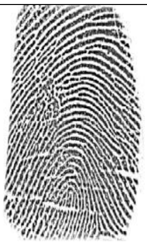

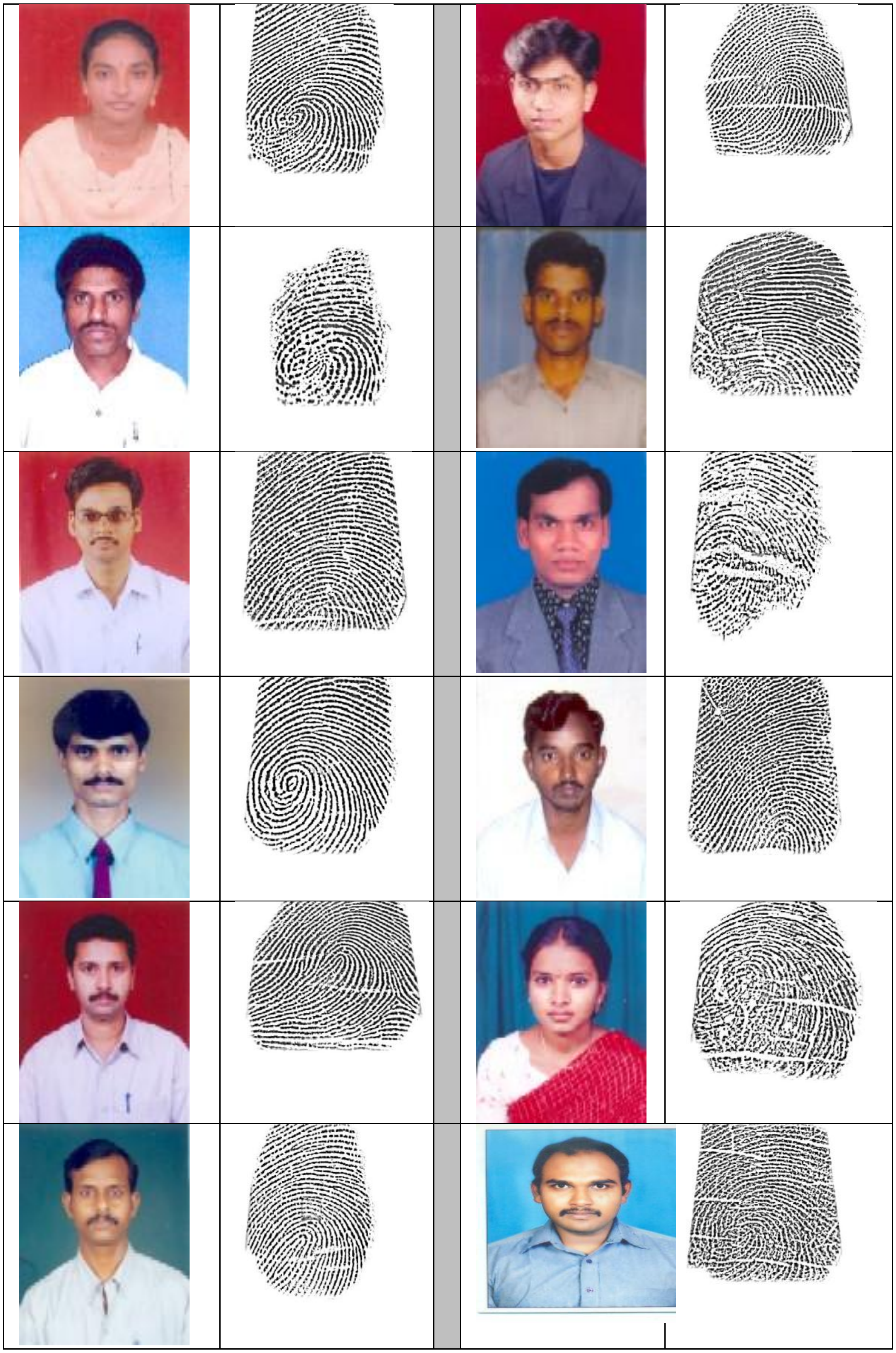

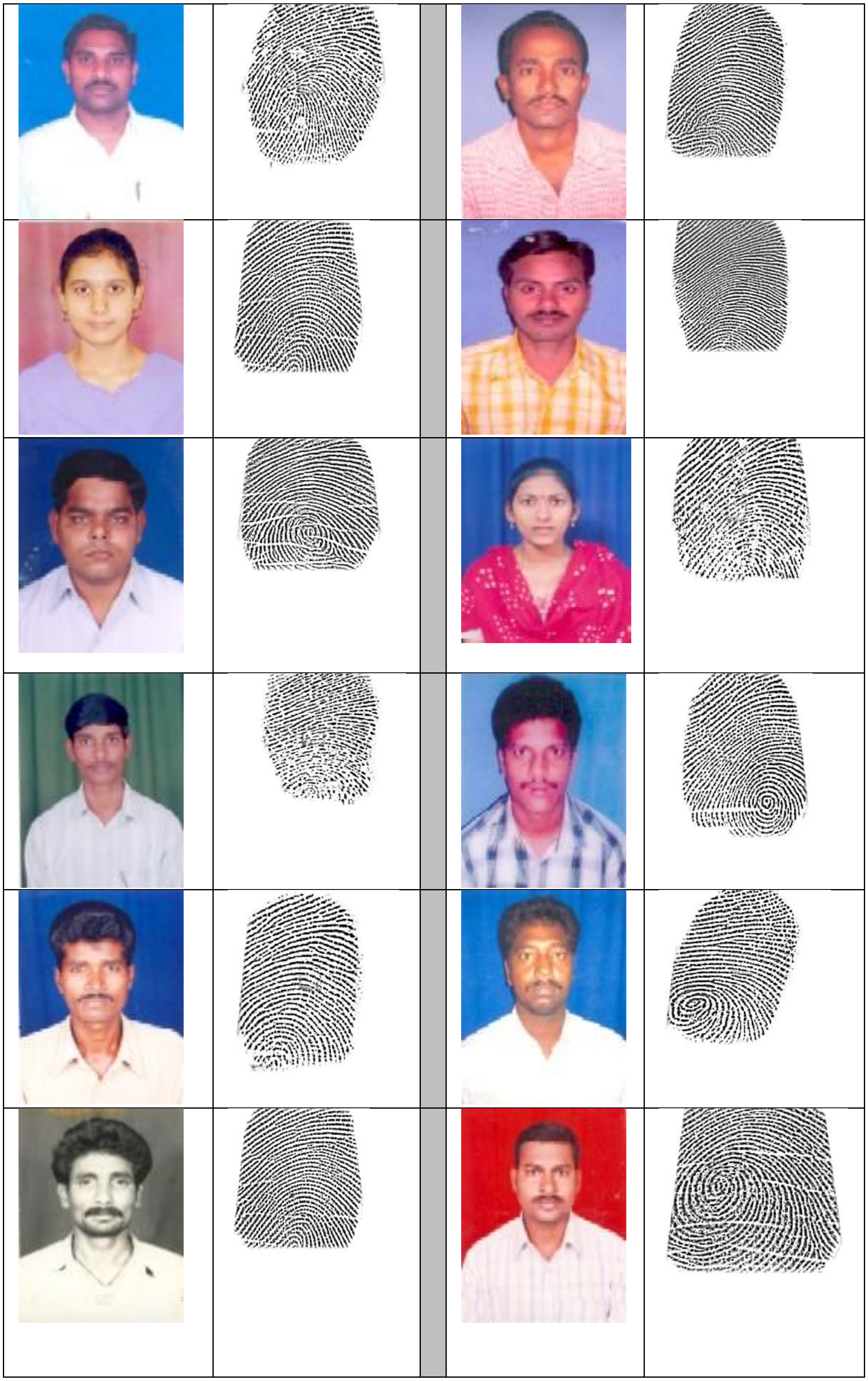

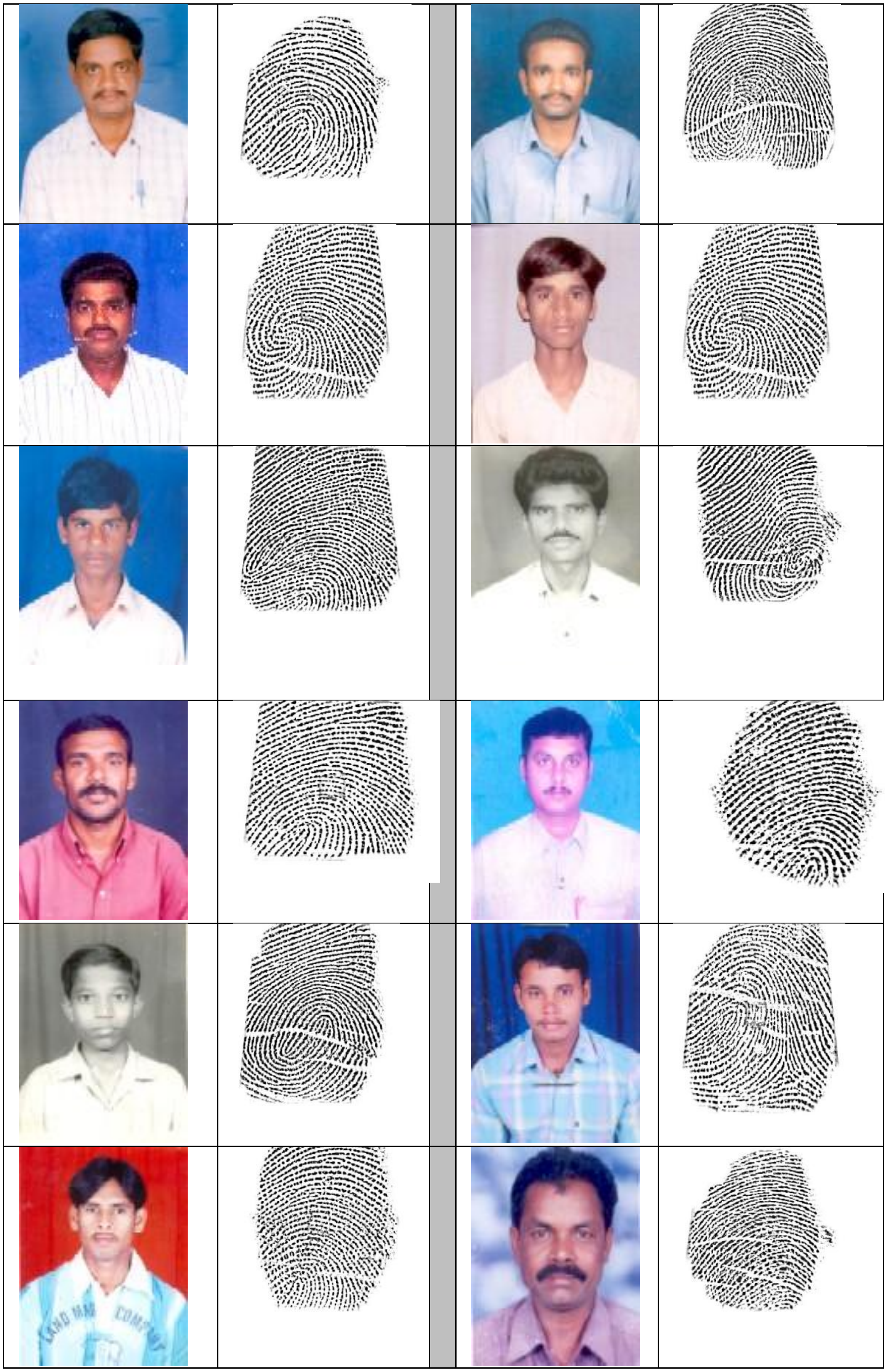

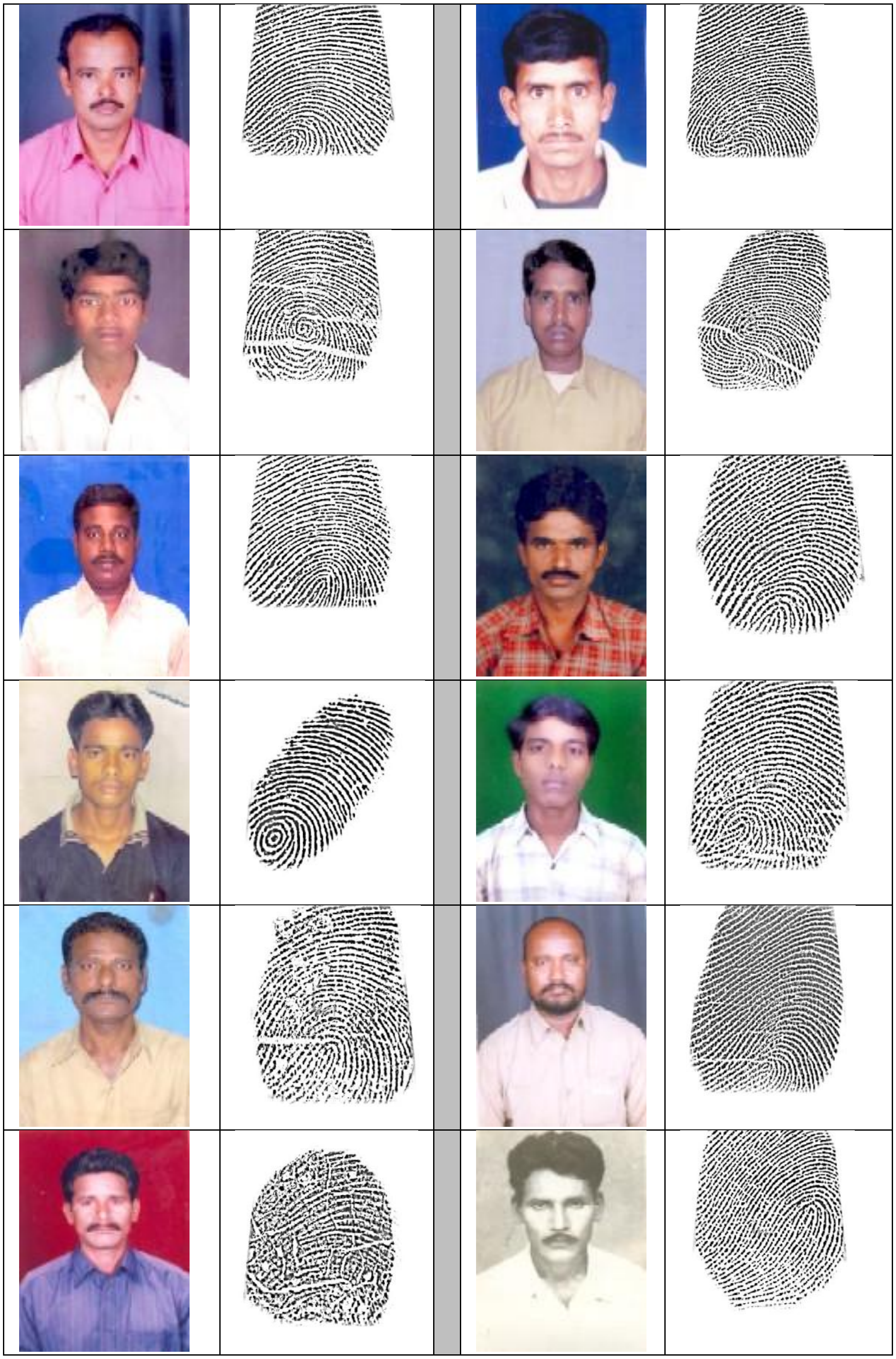

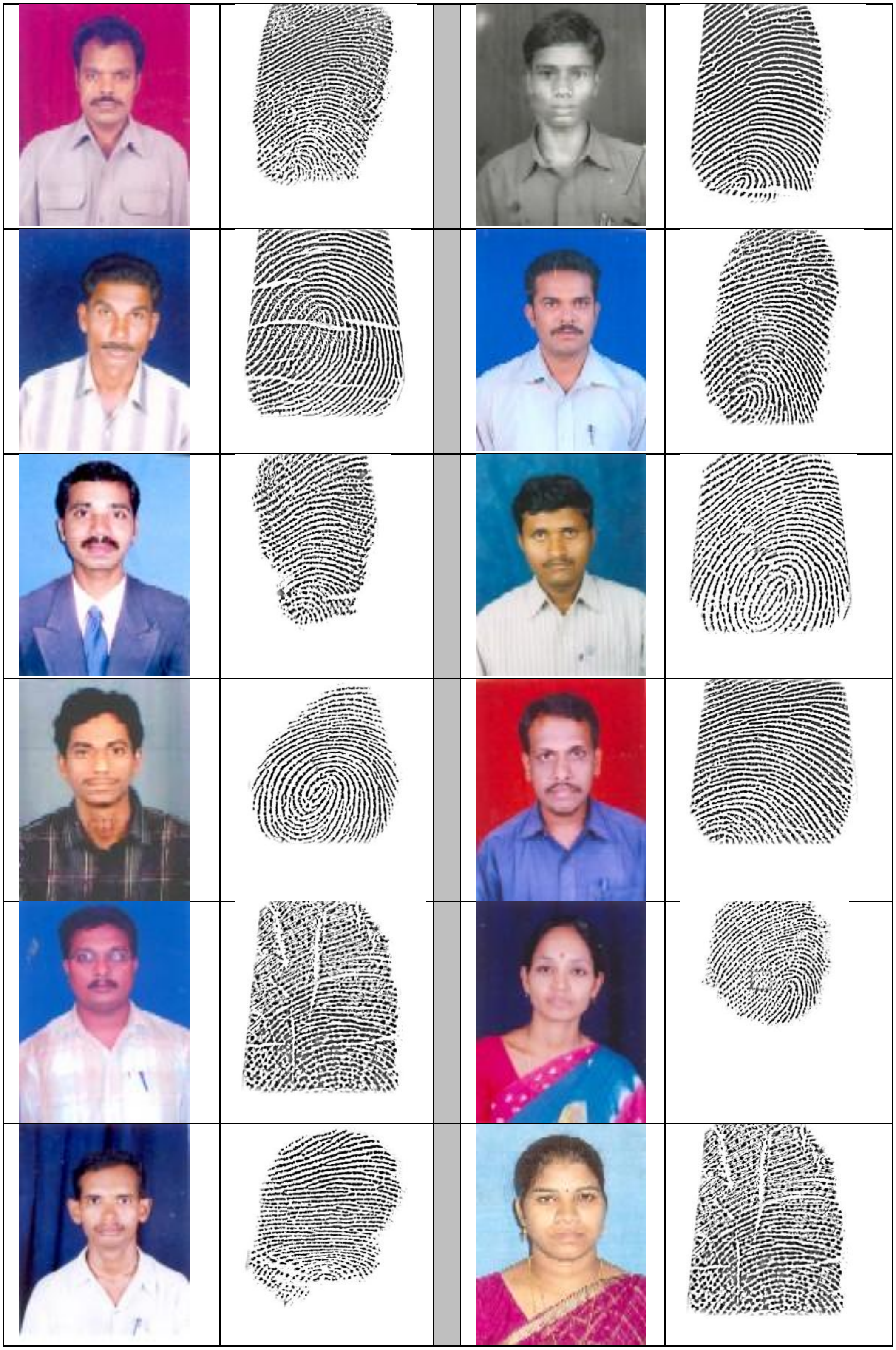

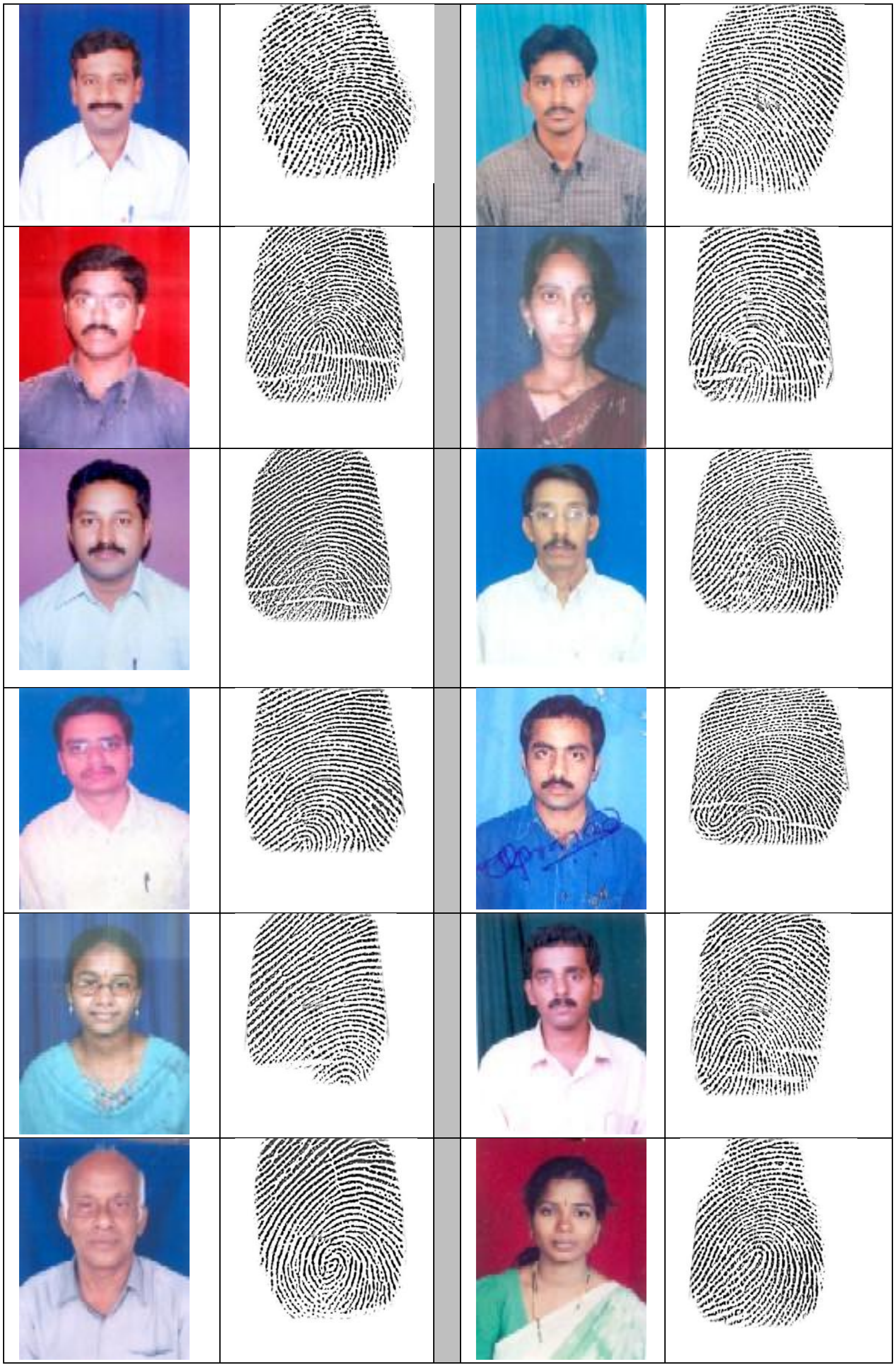

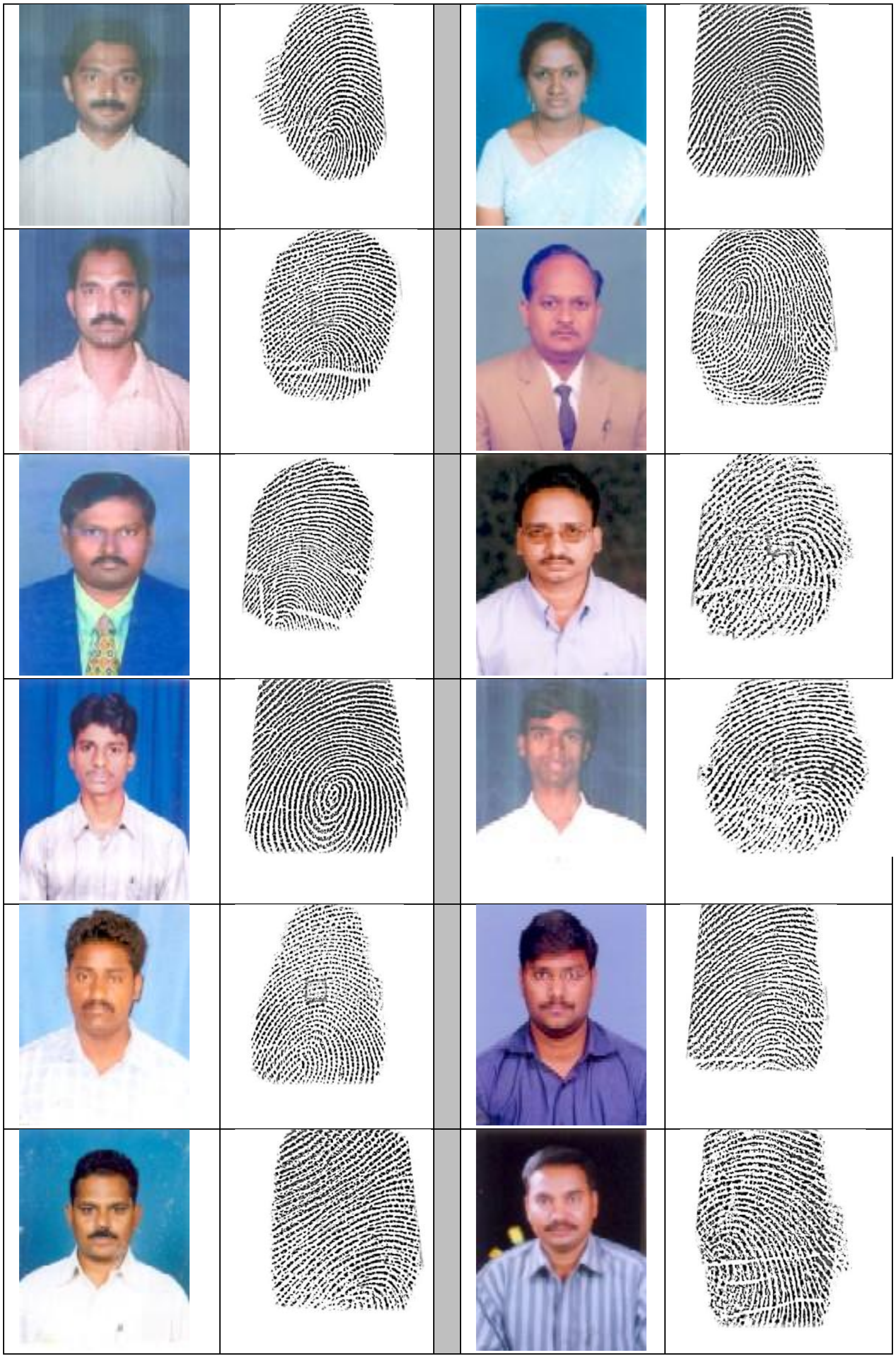

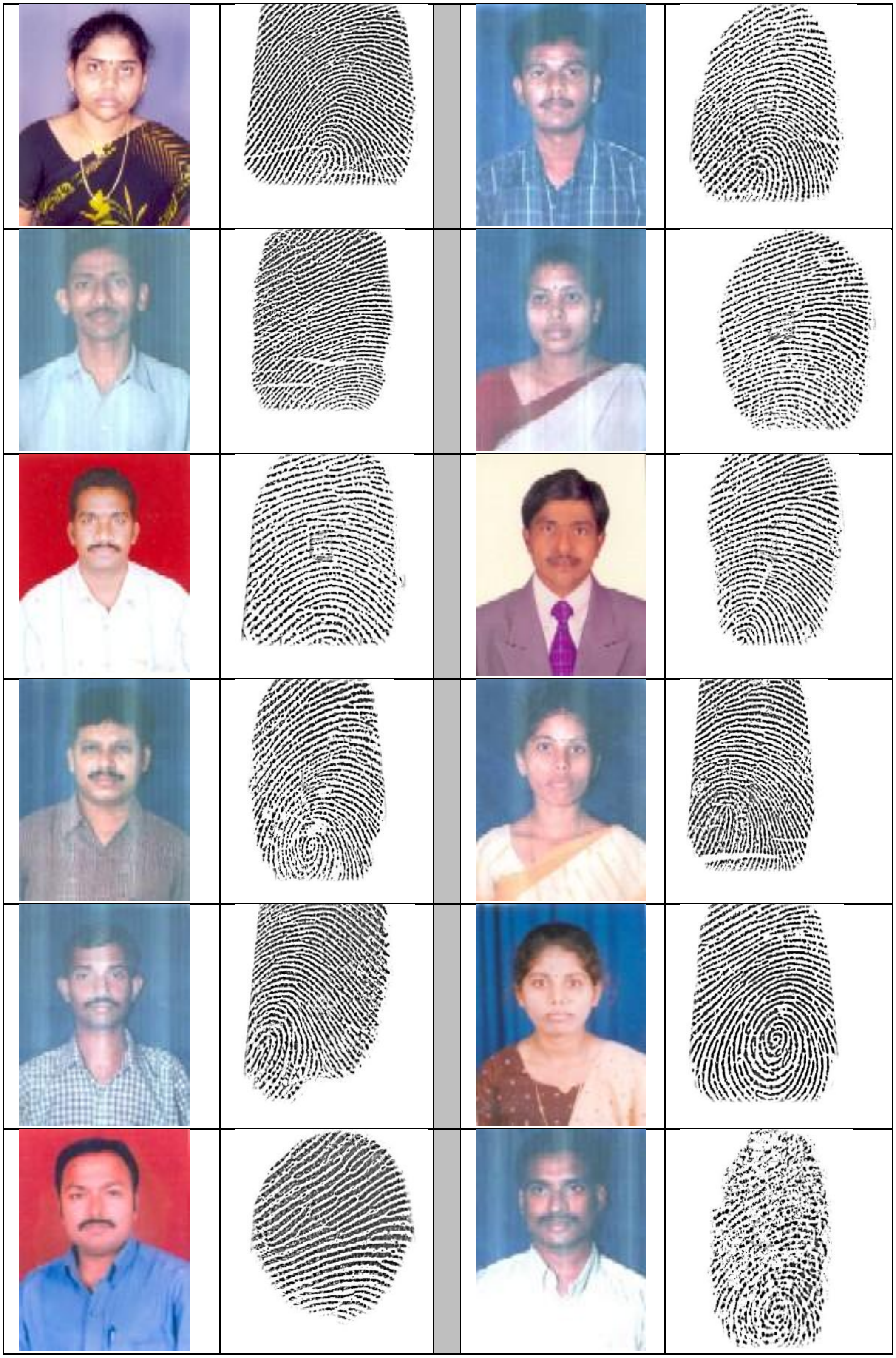

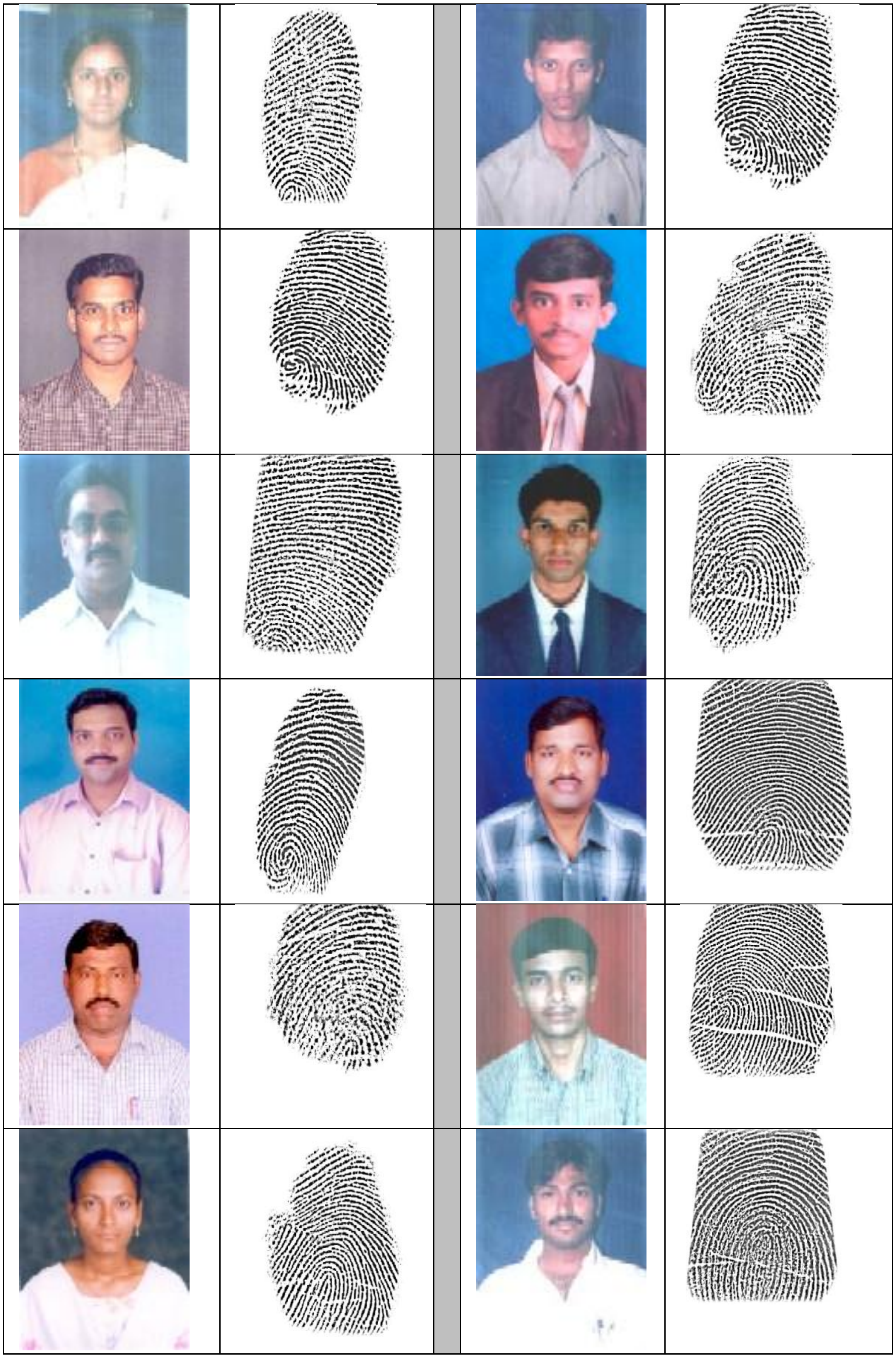

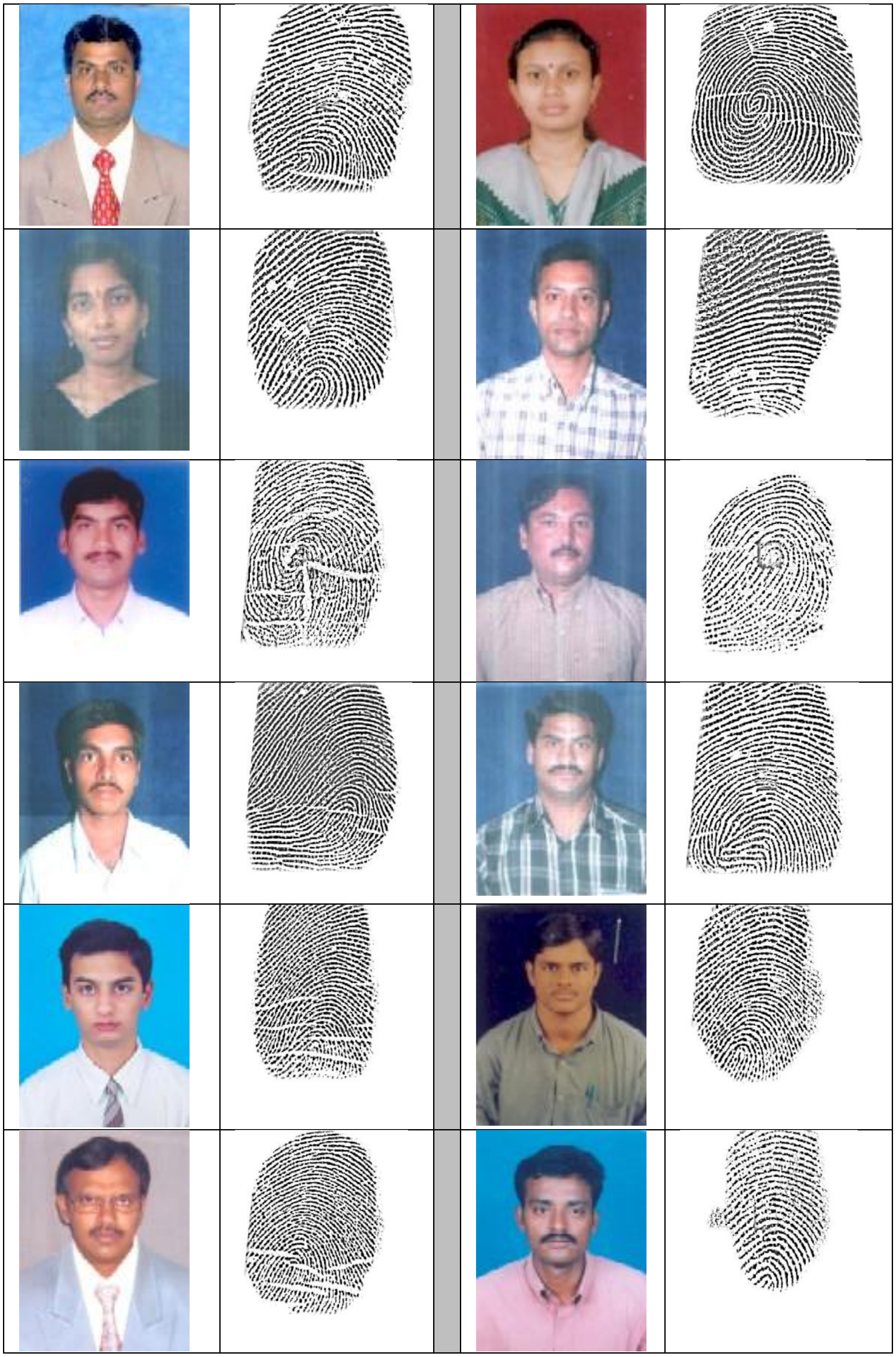

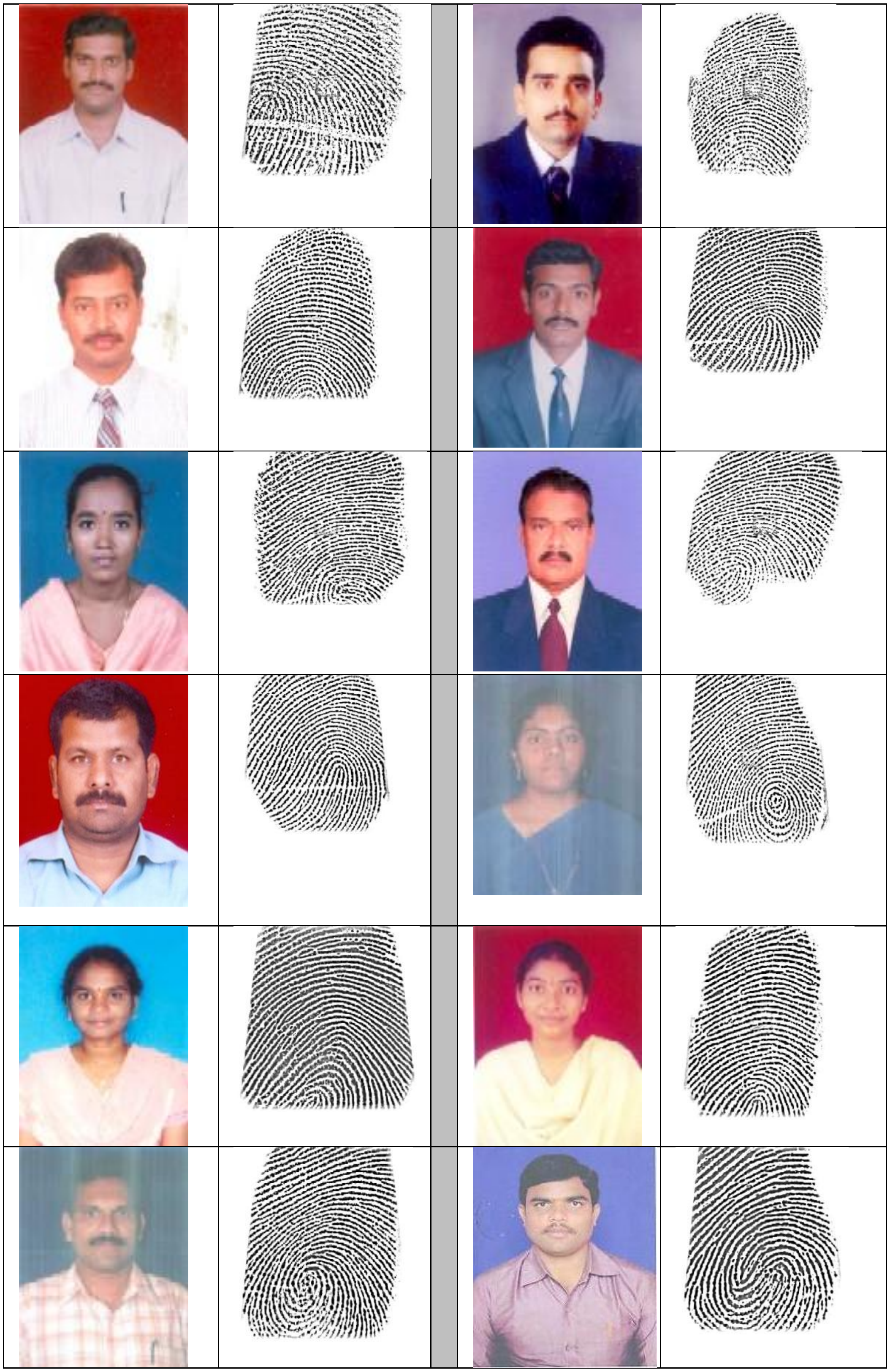

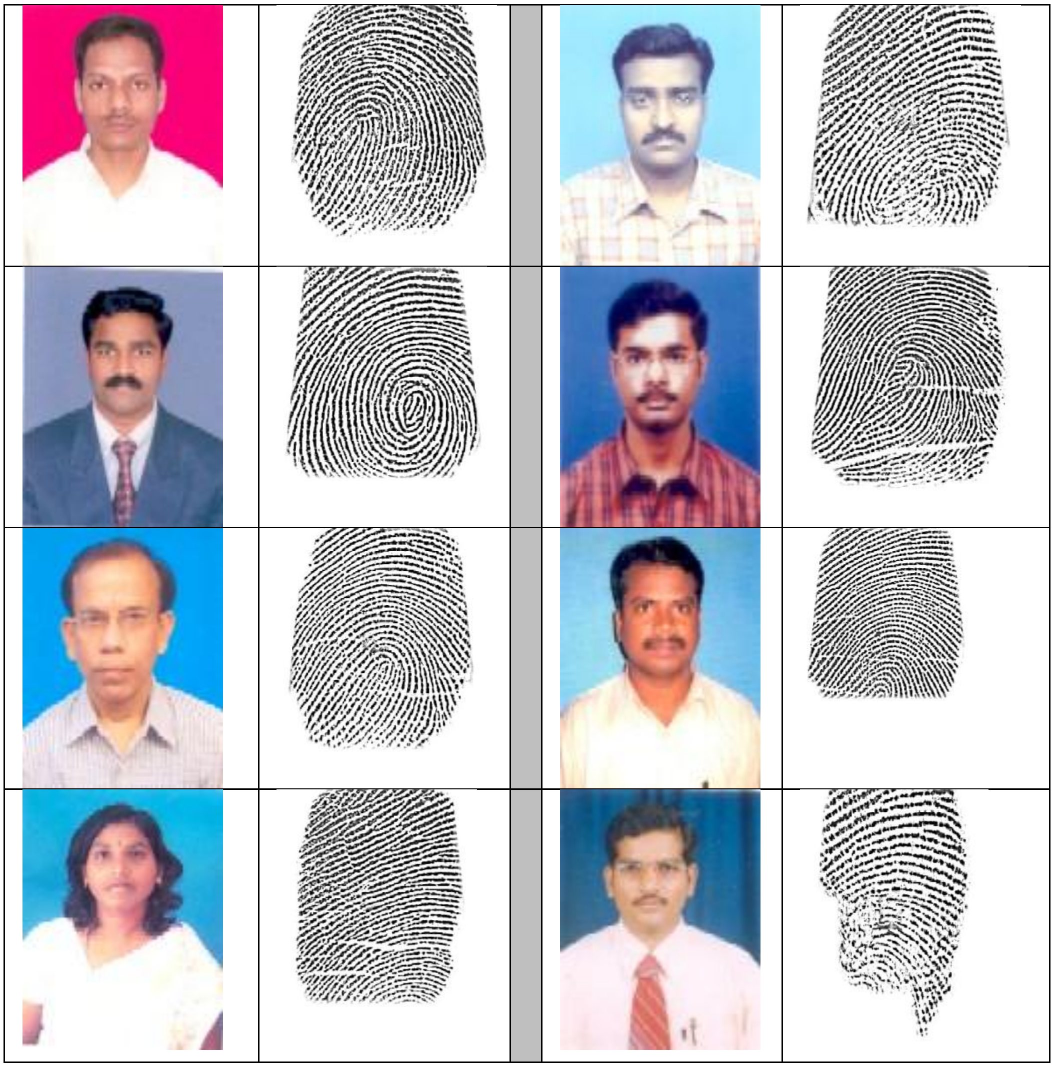

APPENDIX II

http://dx.doi.org/10.17632/r7pbjvdrcb.1

APPENDIX III

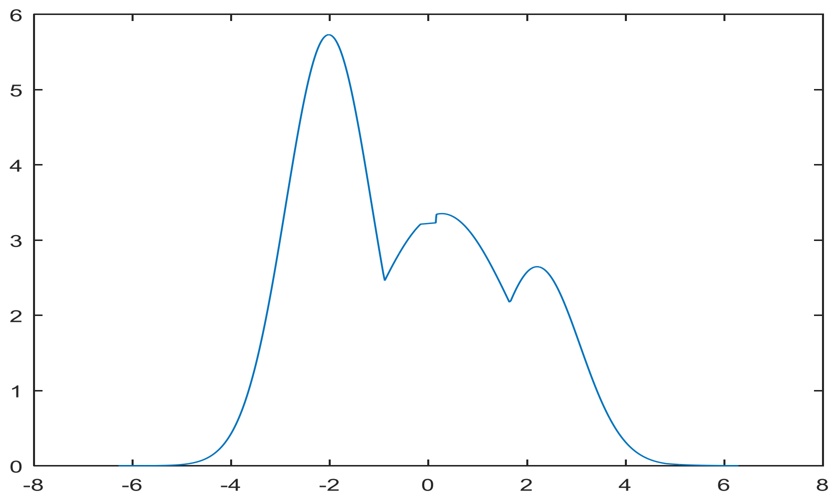

Fig. 8.(a) Fusion with three traits

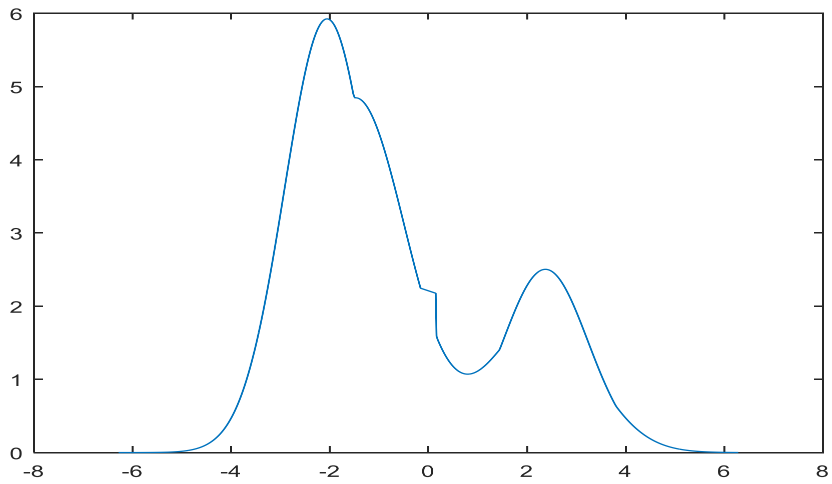

Fig.8.(b) Fusion with three traits

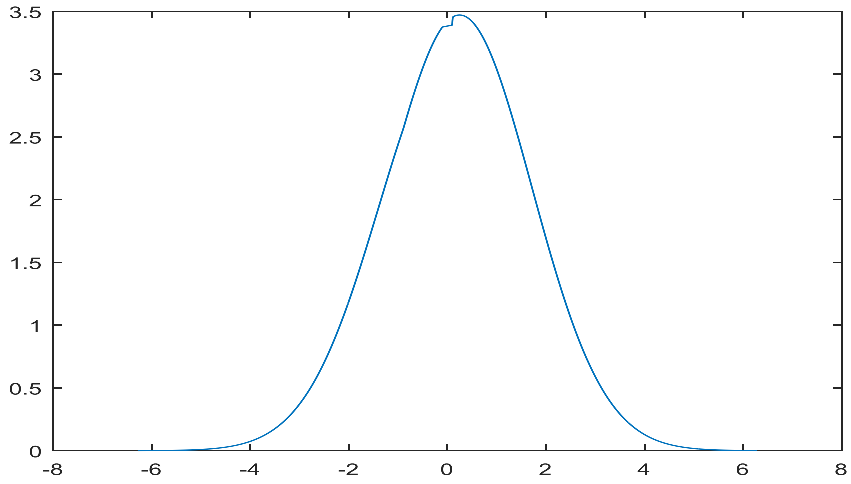

Fig. 8.(c) Fusion with three traits

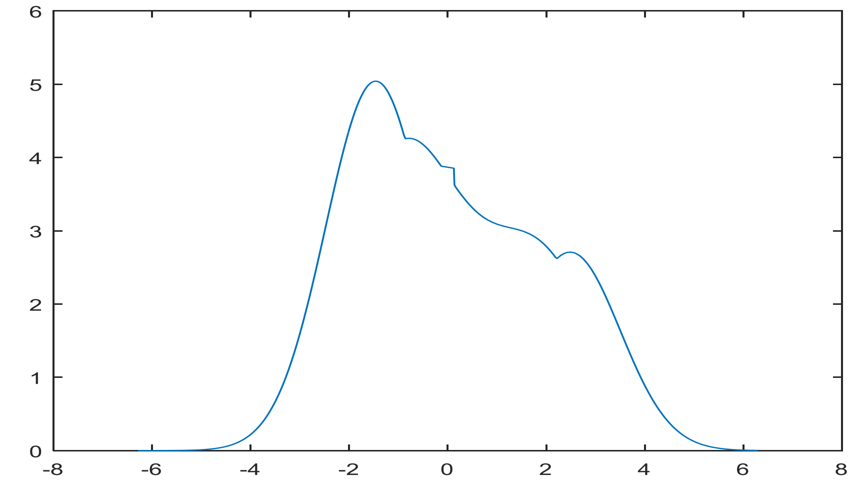

Fig. 8.(d) Fusion with three traits

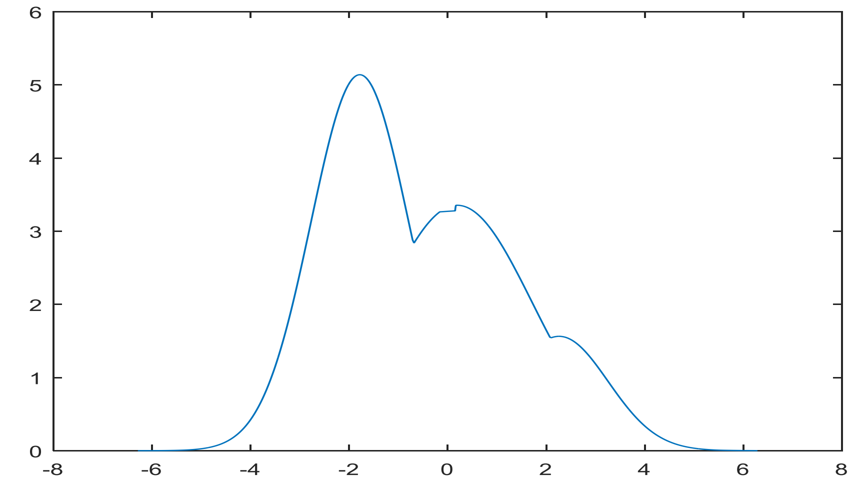

Fig. 8.(e) Fusion with three traits

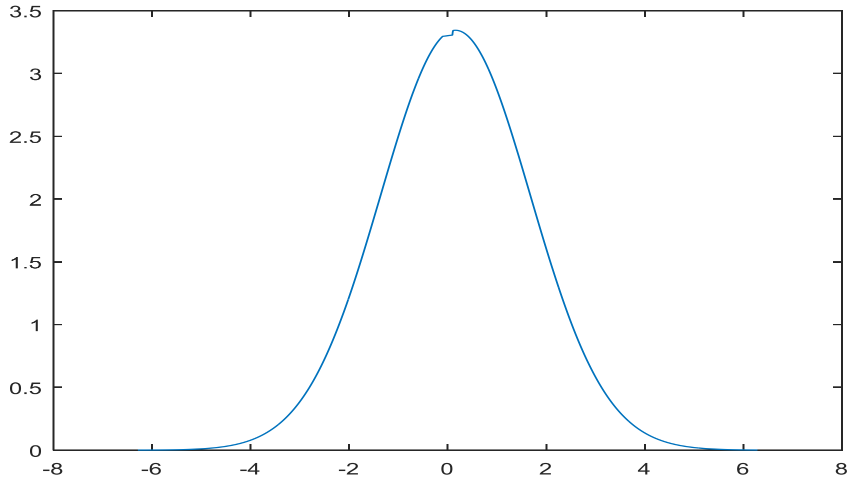

Fig. 8.(f) Fusion with three traits

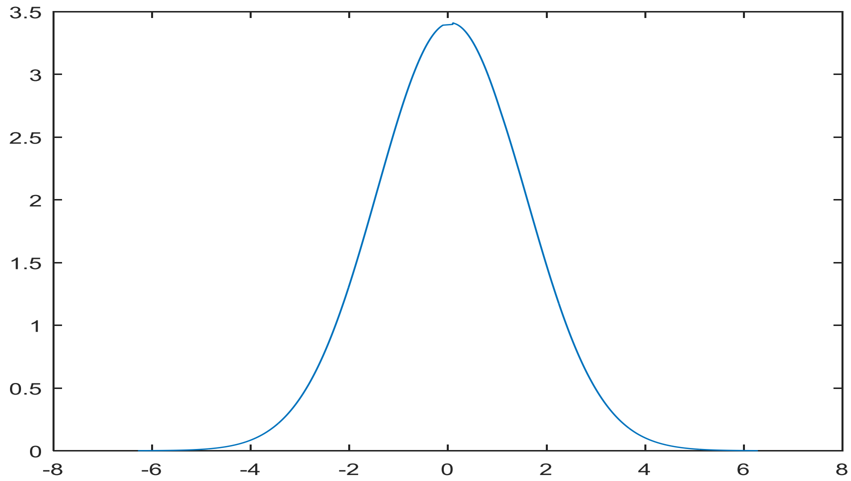

Fig. 8.(g) Fusion with three traits

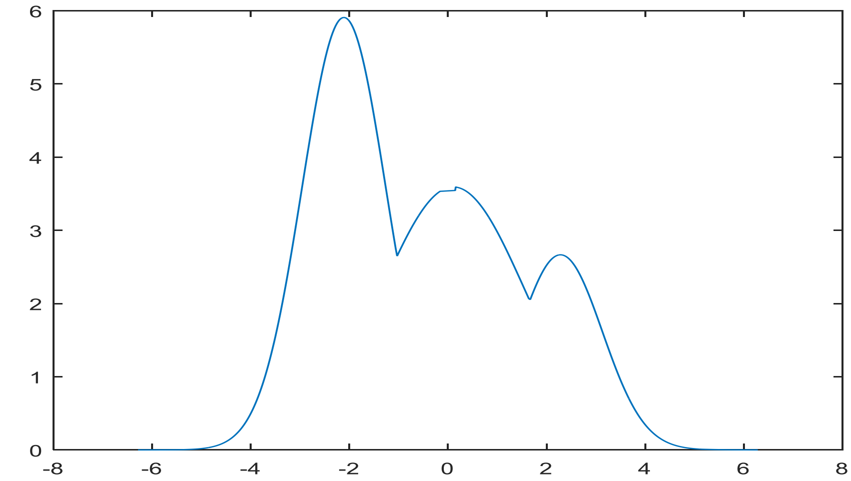

Fig. 8.(h) Fusion with three traits
